# High quality draft genomic sequence of *Arenimonas donghaensis* DSM 18148^T^

**DOI:** 10.1186/s40793-015-0055-4

**Published:** 2015-08-26

**Authors:** Fang Chen, Hui Wang, Yajing Cao, Xiangyang Li, Gejiao Wang

**Affiliations:** State Key Laboratory of Agricultural Microbiology, College of Life Sciences and Technology, Huazhong Agricultural University, Wuhan, 430070 P. R. China

**Keywords:** *Arenimonas*, *Arenimonas donghaensis*, Comparative genomics, Genome sequence, *Xanthomonadaceae*

## Abstract

*Arenimonas donghaensis* is the type species of genus *Arenimonas* which belongs to family *Xanthomonadaceae* within *Gammaproteobacteria*. In this study, a total of five type strains of *Arenimonas* were sequenced. The draft genomic information of *A. donghaensis* DSM 18148^T^ is described and compared with other four genomes of *Arenimonas*. The genome size of *A. donghaensis* DSM 18148^T^ is 2,977,056 bp distributed in 51 contigs, containing 2685 protein-coding genes and 49 RNA genes.

## Introduction

*Arenimonas donghaensis*DSM 18148^T^ (= HO3-R19^T^ = KACC 11381^T^) was isolated from seashore sand [1] which belongs to family *Xanthomonadaceae*. So far, the genus *Arenimonas* contained seven species, *Arenimonas donghaensis* (type species) [1], *Arenimonas malthae* [2], *Arenimonas oryziterrae* [3], *Arenimonas composti* [3], *Arenimonas metalli* [4], *Arenimonas daejeonensis* [5] and *Arenimonas daechungensis* [6]. These bacteria were isolated from seashore sand [1], oil-contaminated soil [2], rice rhizosphere [3], compost [3], iron mine [4], compost [5] and sediment of a eutrophic reservoir [6], respectively. The species *A. composti* [3] was previously classified as *Aspromonas composti* [7].

The common characteristics of the *Arenimonas* strains are Gram-staining-negative, aerobic, rod-shaped, non-spore-forming, oxidase-positive, non-indole-producing, non-nitrate-reducing, containing iso-C_16:0_ and iso-C_15:0_ as the major fatty acids, phosphatidylglycerol and phosphatidylethanolamine as the major polar lipids, Q-8 as the major respiratory quinone, and possessing relatively high DNA G + C content (63.9–70.8 mol %) [1–7].

In order to provide genome information and determine genomic differences of *Arenimonas* species, we performed genome sequencing of strains *A. donghaensis*DSM 18148^T^, *A. composti*KCTC 12666^T^, *A. malthae*CCUG 53596^T^, *A. metalli* CF5-1^T^ and *A. oryziterrae*KCTC 22247^T^*.* In this study, we report the genomic features of *A. donghaensis*DSM 18148^T^ and compare it to the close relatives.

## Organism information

### Classification and features

**Fig. 1:**
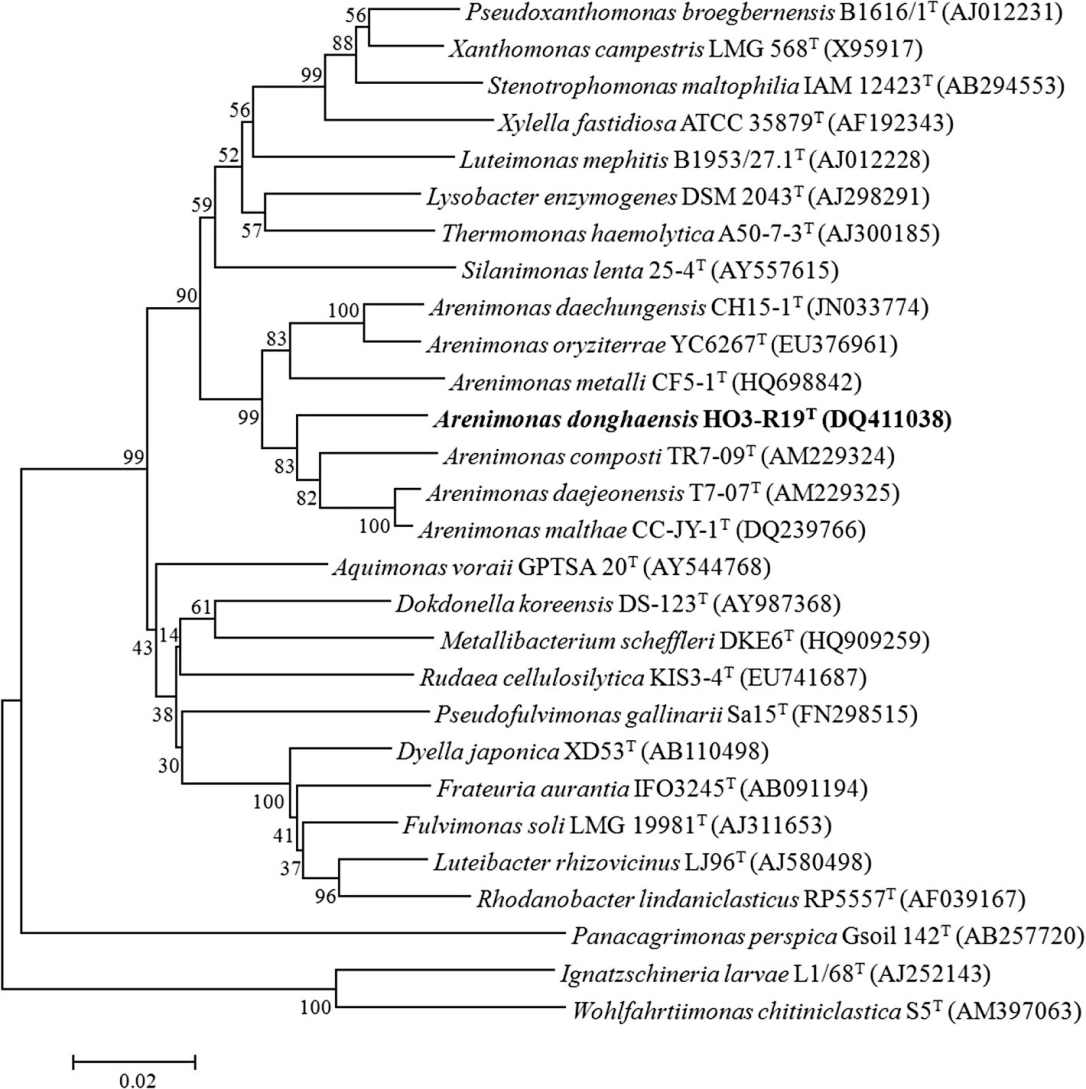
A phylogenetic tree based on the 16S rRNA gene sequences highlighting the position of *A. donghaensis* HO3-R19^T^ (shown in bold) related to the strains of *Arenimonas*. The GenBank accession numbers are shown in parentheses. Sequences were aligned using CLUSTALW, and phylogenetic inferences were obtained using the neighbor-joining method within the MEGA 5.05 software [8]. Numbers at the nodes represent percentages of bootstrap values obtained by repeating the analysis 1000 times to generate a majority consensus tree. The scale bar indicates 0.02 nucleotide change per nucleotide position

Strain *A. donghaensis*DSM 18148^T^ shares 93.1–95.7 % 16S rRNA gene identities with the other six type strains of *Arenimonas* species, *A. malthae* CC-JY-1^T^ (DQ239766) (95.7 %), *A. daejeonensis* T7-07^T^ (AM229325) (95.7 %), *A. metalli* CF5-1^T^ (HQ698842) (94.6 %), *A. oryziterrae* YC6267^T^ (EU376961) (94.3 %), *A. composti* TR7-09^T^ (AM229324) (94.3 %) and *A. daechungensis* CH15-1^T^ (JN033774) (93.1 %). A 16S rRNA gene based neighbor-joining phylogenetic tree of the related strains was obtained using MEGA 5.05 software [8] (Fig. 1).

**Fig. 2:**
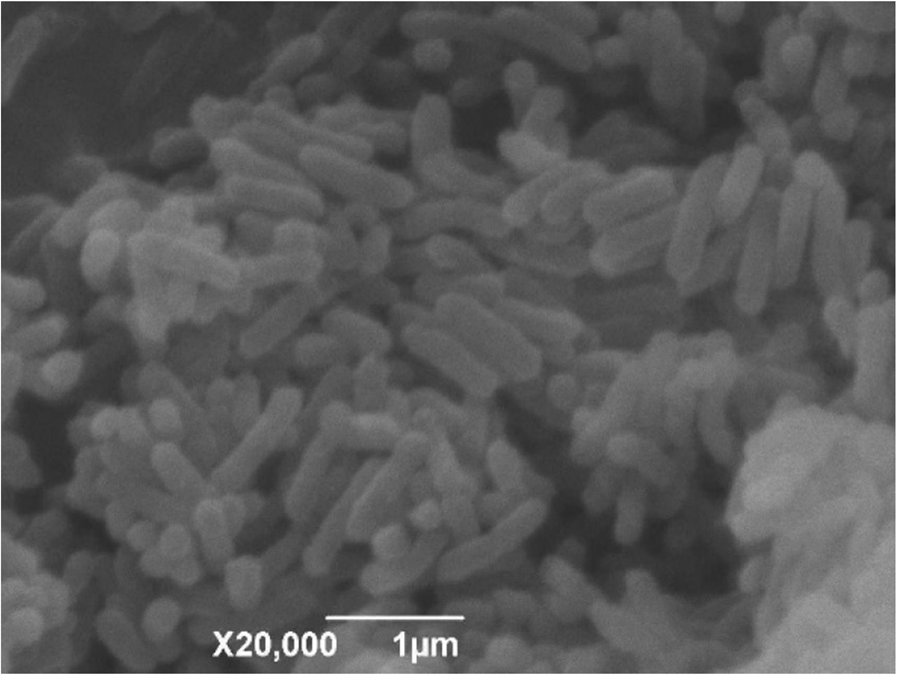
A scanning electron micrograph of *A. donghaensis* DSM 18148^T^ cells

Cells of *A. donghaensis*DSM 18148^T^ are Gram-negative, aerobic, non-spore-forming, straight or slightly curved rods, motile by means of a single polar flagellum. Colonies are yellowish white, translucent and convex on R2A agar after 3 d cultivation (Fig. 2). API ID 32 GN and Biolog GN2 MicroPlate systems (bioMe’rieux) were used to investigate sole carbon source utilization, and β-hydroxybutyric acid, L-alaninamide, L-glutamic acid and glycyl-L-glutamic acid could be utilized by strain DSM 18148^T^ (Table 1).

**Table 1:** Classification and general features of *A. donghaensis* strain DSM 18148^T^ according to the MIGS recommendations [21]

MIGS ID	Property	Term	Evidence code^a^
	Classification	Domain *Bacteria*	TAS [22]
		Phylum *Proteobacteria*	TAS [23]
		Class *Gammaproteobacteria*	TAS [24, 25]
		Order *Xanthomonadales*	TAS [24, 26]
		Family *Xanthomonadaceae*	TAS [24, 26]
		Genus *Arenimonas*	TAS [1]
		Species *Arenimonas donghaensis*	TAS [1]
		Type strain: HO3-R19^T^ (= KACC 11381^T^ = DSM 18148^T^)	
	Gram stain	negative	TAS [1]
	Cell shape	straight or slightly curved rod	TAS [1]
	Motility	motile	TAS [1]
	Sporulation	non-spore-forming	TAS [1]
	Temperature range	4–37 °C	TAS [1]
	Optimum temperature	28 °C	TAS [1]
	pH range; Optimum	7.0–9.0; 8.0	TAS [1]
	Carbon source	casein, tyrosine and gelatin; β-hydroxybutyric acid, L-alaninamide, L-glutamic acid and glycyl-L-glutamic acid	IDA
GS-6	Habitat	seashore sand	TAS [1]
MIGS-6.3	Salinity	0–3 % NaCl (w/v)	TAS [1]
MIGS-22	Oxygen requirement	aerobic	TAS [1]
MIGS-15	Biotic relationship	free-living	NAS
MIGS-14	Pathogenicity	non-pathogen	NAS
MIGS-4	Geographic location	Pohang city, Korea	TAS [1]
MIGS-5	Sample collection	not reported	
MIGS-4.1	Latitude	not reported	
MIGS-4.2	Longitude	not reported	
MIGS-4.4	Altitude	not reported	

a,Evidence codes – *IDA* Inferred from Direct Assay, *TAS* Traceable Author Statement (i.e., a direct report exists in the literature), *NAS* Non-traceable Author Statement (i.e., not directly observed for the living, isolated sample, but based on a generally accepted property for the species, or anecdotal evidence). These evidence codes are from the Gene Ontology project [27]

The major fatty acids of *A. donghaensis*DSM 18148^T^ are iso-branched types, such as iso-C_16:0_, iso-C_15:0_ and iso-C_17:1_*ω*9*c* [1]. Major isoprenoid quinone of this bacterium is Q-8 [1]. Diphosphatidylglycerol (DPG), PG and PE are the major polar lipids of this strain [1].

## Genome sequencing information

### Genome project history

Genome sequencing project of *A. donghaensis*DSM 18148^T^ was carried out in April, 2013 and was finished in two months. The obtained high-quality draft genome of *A. donghaensis*DSM 18148^T^ has been deposited at DDBJ/EMBL/GenBank under accession number AVCJ00000000. The version described in this study is the first version, AVCJ01000000. The genome sequencing project information is summarized in Table 2.

**Table 2:** Project information

MIGS ID	Property	Term
MIGS 31	Finishing quality	High-quality draft
MIGS-28	Libraries used	Illumina Paired-End library (300 bp insert size)
MIGS 29	Sequencing platforms	Illumina Hiseq2000
MIGS 31.2	Fold coverage	332.4×
MIGS 30	Assemblers	SOAPdenovo v1.05
MIGS 32	Gene calling method	GeneMarkS+
	Locus Tag	N788
	GenBank ID	AVCJ00000000
	GenBank Date of Release	2014/08/25
	GOLD ID	Gi0067066
	BIOPROJECT	PRJNA214575
MIGS 13	Source Material Identifier	DSM 18148
	Project relevance	Genome comparison

### Growth conditions and genomic DNA preparation

*A. donghaensis*DSM 18148^T^ was cultivated aerobically in LB medium at 28 °C for 3 d. The DNA was extracted, concentrated and purified using the QiAamp kit according to the manufacturer’s instruction (Qiagen, Germany).

### Genome sequencing and assembly

The whole-genome sequence of *A. donghaensis*DSM 18148^T^ was determined using the Illumina Hiseq2000 [9] with the Paired-End library strategy (300 bp insert size) at Shanghai Majorbio Bio-pharm Technology Co., Ltd. [10] (Shanghai, China). A total of 9,571,421 reads with an average read length of 93 bp (885.9 Mb data) was obtained. The detailed methods of library construction and sequencing can be found at Illumina’s official website [9]. Using SOAPdenovo v1.05 [11], these reads were assembled into 51 contigs (>200 bp) with a genome size of 2,977,056 bp and an average coverage of 332.4 x.

### Genome annotation

The draft sequence of strain *A. donghaensis*DSM 18148^T^ was submitted to NCBI Prokaryotic Genome Annotation Pipeline [12] for annotation according to the draft WGS annotation guideline at this website. This annotation pipeline combines the GeneMarkS+ algorithm with the similarity-based gene detection approach to calling gene. The function of the predicted genes from the automatic result was manually modified through BlastX analysis against the NCBI protein database with E-value cutoff 1-e^20^.

## Genome properties

**Fig. 3:**
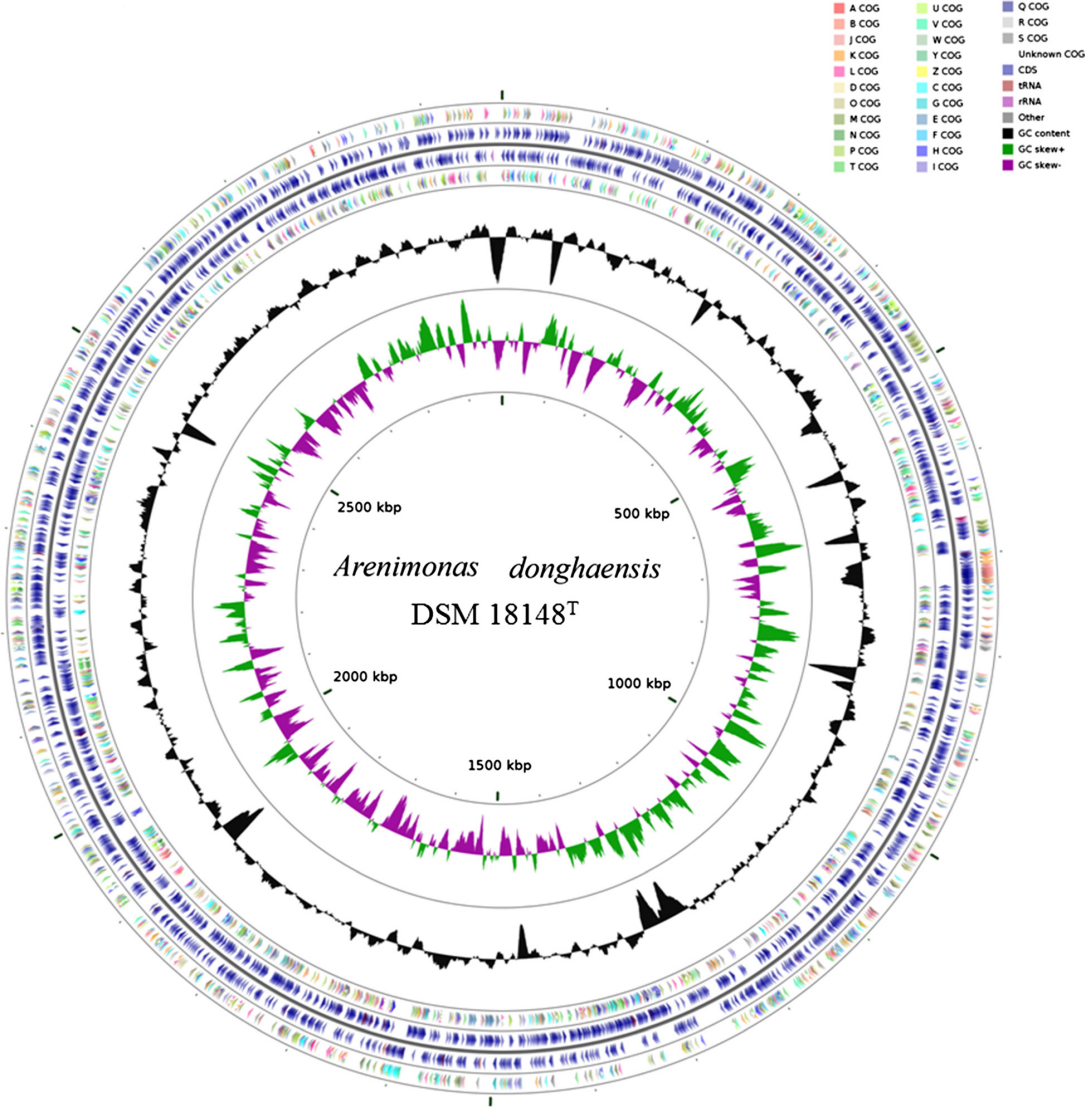
Graphical circular map of *A. donghaensis* DSM 18148^T^ genome. From outside to center, ring 1, 4 show protein-coding genes colored by COG categories on forward/reverse strand; ring 2, 3 denote genes on forward/reverse strand; ring 5 shows G + C% content plot, and the innermost ring shows GC skew

The whole genome of *A. donghaensis*DSM 18148^T^ is 2,977,056 bp in length, with a G + C content of 68.7 % (Fig. 3 and Table 3), and distributed in 51 contigs (>200 bp). Of the 2735 predicted genes, 2685 (98.17 %) are protein-coding genes, 49 (1.79 %) are RNA genes and 1 (0.04 %) are pseudogenes. A total of 472 (17.26 %) CDSs were assigned with putative functions, while the remaining ones were annotated as hypothetical proteins. The result of protein function classification is shown in Table 4, which was performed by searching all the predicted coding sequences of strain DSM 18148^T^ against the Clusters of Orthologous Groups protein database [13] using BlastP algorithm with E-value cutoff 1-e^10^. A more detailed summary of the genome properties about this strain is provided in Table 3.

**Table 3:** Genome statistics

Attribute	Value	% of Total
Genome size (bp)	2,977,056	100.00
DNA coding (bp)	2,722,012	91.43
DNA G + C (bp)	2,046,559	68.74
DNA scaffolds	49	
Total genes	2735	100.00
Protein coding genes	2685	98.17
RNA genes	49	1.79
Pseudo genes	1	0.04
Genes in internal clusters		
Genes with function prediction	472	17.26
Genes assigned to COGs	2244	82.05
Genes with Pfam domains	2194	80.22
Genes with signal peptides	362	13.24
Genes with transmembrane helices	717	26.22
CRISPR repeats	0	0.00

**Table 4:** Number of genes associated with general COG functional categories

Code	Value	% age	Description
J	163	6.07	Translation, ribosomal structure and biogenesis
A	1	0.04	RNA processing and modification
K	127	4.73	Transcription
L	107	3.99	Replication, recombination and repair
B	1	0.04	Chromatin structure and dynamics
D	28	1.04	Cell cycle control, Cell division, chromosome partitioning
V	56	2.09	Defense mechanisms
T	171	6.37	Signal transduction mechanisms
M	155	5.77	Cell wall/membrane biogenesis
N	37	1.38	Cell motility
U	68	2.53	Intracellular trafficking and secretion
O	114	4.25	Posttranslational modification, protein turnover, chaperones
C	146	5.44	Energy production and conversion
G	57	2.12	Carbohydrate transport and metabolism
E	173	6.44	Amino acid transport and metabolism
F	55	2.05	Nucleotide transport and metabolism
H	111	4.13	Coenzyme transport and metabolism
I	102	3.80	Lipid transport and metabolism
P	96	3.58	Inorganic ion transport and metabolism
Q	49	1.82	Secondary metabolites biosynthesis, transport and catabolism
R	241	8.98	General function prediction only
S	186	6.93	Function unknown
-	441	16.42	Not in COGs

The total is based on the total number of protein coding genes in the genome

## Insights from the genome sequences

Strain *A. donghaensis*DSM 18148^T^ can only use several sole carbon sources and cannot assimilate glucose and other sugars [1]. Genome analysis using the Kyoto Encyclopedia of Genes and Genomes (KEGG) [14] orthology and pathway assignment analysis revealed this strain has a complete TCA cycle, but lacks the hexokinase which catalyzes the first step of glycolysis, as well as the glucose-6-phosphate dehydrogenase, gluconolactonase and 6-phosphogluconate dehydrogenase that responsible for the oxidative phase of pentose phosphate pathway. This is in agreement with the experimental result that this bacterium can only use several sole carbon sources.

**Fig. 4:**
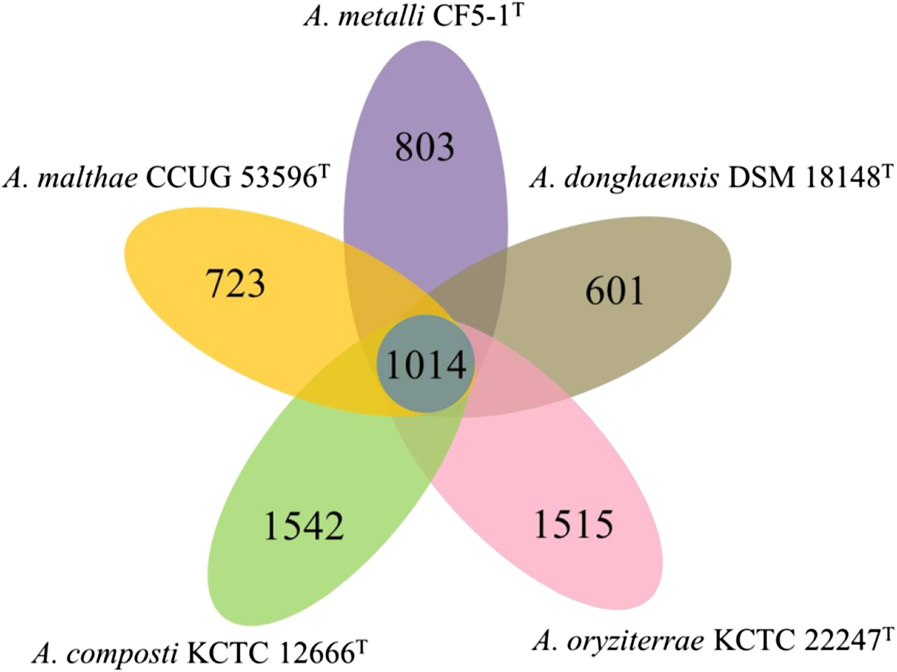
Genome comparison among the five *Arenimonas* species. Venn diagram illustrates the number of genes unique or shared among the five *Arenimonas* genomes

The general features of the five *Arenimonas* sequenced genomes are summarized in Table 5. Orthologs clustering analysis was performed using OrthoMCL [15] with Match cutoff of 50 % and E-value Exponent cutoff of 1-e^5^ for the five *Arenimonas* genomes. These five *Arenimonas* bacteria share 1014 genes, which are classified into 21 COG functional categories. The major categories are energy production and conversion (8.7 %), amino acid transport and metabolism (8.7 %), coenzyme transport and metabolism (5.8 %), lipid transport and metabolism (5.1 %), translation, ribosomal structure and biogenesis (12.4 %), replication, recombination and repair (5.2 %), cell wall/membrane/envelope biogenesis (5.9 %), posttranslational modification, protein turnover, chaperones (6.3 %), general function prediction only (8.4 %), function unknown (7.3 %) and signal transduction mechanisms (5.3 %) (Fig. 4 and Table 6).

**Table 5:** General features of the five *Arenimonas* genomes

Strains	Source	Size (Mb)	CDSs	rRNA clusters	tRNAs	Draft/finished	Genome status contigs	Contigs N50	GenBank no.
*A. composti* KCTC 12666^T^	Compost	3.16	2849	3	45	Draft	95	81,415	AWXU00000000
*A. donghaensis* DSM 18148^T^	Seashore sand	2.98	2685	4	45	Draft	51	159,562	AVCJ00000000
*A. malthae* CCUG 53596^T^	Oil-contaminated soil	3.11	2861	5	44	Draft	221	29,626	AVCH00000000
*A. metalli* CF5-1^T^	Iron mine	3.06	2775	2	44	Draft	65	99,300	AVCK00000000
*A. oryziterrae* KCTC 22247^T^	Rice rhizosphere	3.09	2897	3	45	Draft	45	441,364	AVCI00000000

**Table 6:** Number of genes in the core genome of the five analyzed *Arenimonas* genomes associated with general COG functional categories

Code	Value	% age	Description
A	1	0.10	RNA processing and modification
C	88	8.68	Energy production and conversion
D	16	1.58	Cell cycle control, cell division, chromosome partitioning
E	88	8.68	Amino acid transport and metabolism
F	42	4.14	Nucleotide transport and metabolism
G	20	1.97	Carbohydrate transport and metabolism
H	59	5.82	Coenzyme transport and metabolism
I	52	5.13	Lipid transport and metabolism
J	126	12.43	Translation, ribosomal structure and biogenesis
K	44	4.34	Transcription
L	53	5.23	Replication, recombination and repair
M	60	5.92	Cell wall/membrane/envelope biogenesis
N	12	1.18	Cell motility
O	64	6.31	Posttranslational modification, protein turnover, chaperones
P	32	3.16	Inorganic ion transport and metabolism
Q	22	2.17	Secondary metabolites biosynthesis, transport and catabolism
R	85	8.38	General function prediction only
S	74	7.30	Function unknown
T	54	5.33	Signal transduction mechanisms
U	27	2.66	Intracellular trafficking, secretion, and vesicular transport
V	11	1.08	Defense mechanisms
-	0	0.00	Not in COGs

The total is based on the total number of protein coding genes in the core genome

There are 601 strain-specific genes for *A. donghaensis*DSM 18148^T^ which may contribute to species-specific features of this bacterium. Among them, 359 are classified into 20 COG functional categories major belonging to transcription (6.3 %), general function prediction only (8.5 %), function unknown (7.3 %) and signal transduction mechanisms (9.0 %). The remaining 242 unique genes (40.3 %) are not classified into any COG categories (Fig. 4 and Table 7). In addition, the five *Arenimonas* strains had a pan-genome [16] size of 7501 genes. The nucleotide diversity (π) was calculated using MAUVE v2.3 [17] and DnaSP v5 [18]. The five genomes of *Arenimonas* species had a nucleotide diversity (π) value of 0.18, which means an approximate genus-wide nucleotide sequence homology of 82 %.

**Table 7:** Number of strain-specific genes of *A. donghaensis* DSM 18148^T^ associated with general COG functional categories

Code	Value	% age	Description
C	15	2.50	Energy production and conversion
D	3	0.50	Cell cycle control, cell division, chromosome partitioning
E	17	2.83	Amino acid transport and metabolism
F	3	0.50	Nucleotide transport and metabolism
G	6	1.00	Carbohydrate transport and metabolism
H	15	2.50	Coenzyme transport and metabolism
I	9	1.50	Lipid transport and metabolism
J	7	1.16	Translation, ribosomal structure and biogenesis
K	38	6.32	Transcription
L	11	1.83	Replication, recombination and repair
M	25	4.16	Cell wall/membrane/envelope biogenesis
N	4	0.67	Cell motility
O	10	1.66	Posttranslational modification, protein turnover, chaperones
P	18	3.00	Inorganic ion transport and metabolism
Q	6	1.00	Secondary metabolites biosynthesis, transport and catabolism
R	51	8.49	General function prediction only
S	44	7.32	Function unknown
T	54	8.99	Signal transduction mechanisms
U	7	1.16	Intracellular trafficking, secretion, and vesicular transport
V	16	2.66	Defense mechanisms
-	242	40.27	Not in COGs

The total is based on the total number of strain-specific genes of *A. donghaensis* DSM 18148^T^

The clustered regularly interspaced short palindromic repeats (CRISPRs) mediate resistance to foreign genetic material and thus inhibit horizontal gene transfer [19]. Screening the CRISPRs system in the five *Arenimonas* genomes using CRISPRfinder program online [20] found that only one CRISPR system (on contig 41) exist in the genome of *A. composti*KCTC 12666^T^. This CRISPR length is 5331 bp, with 29 bp direct repeat (DR) sequences be separated by 87 spacers.

**Fig. 5:**
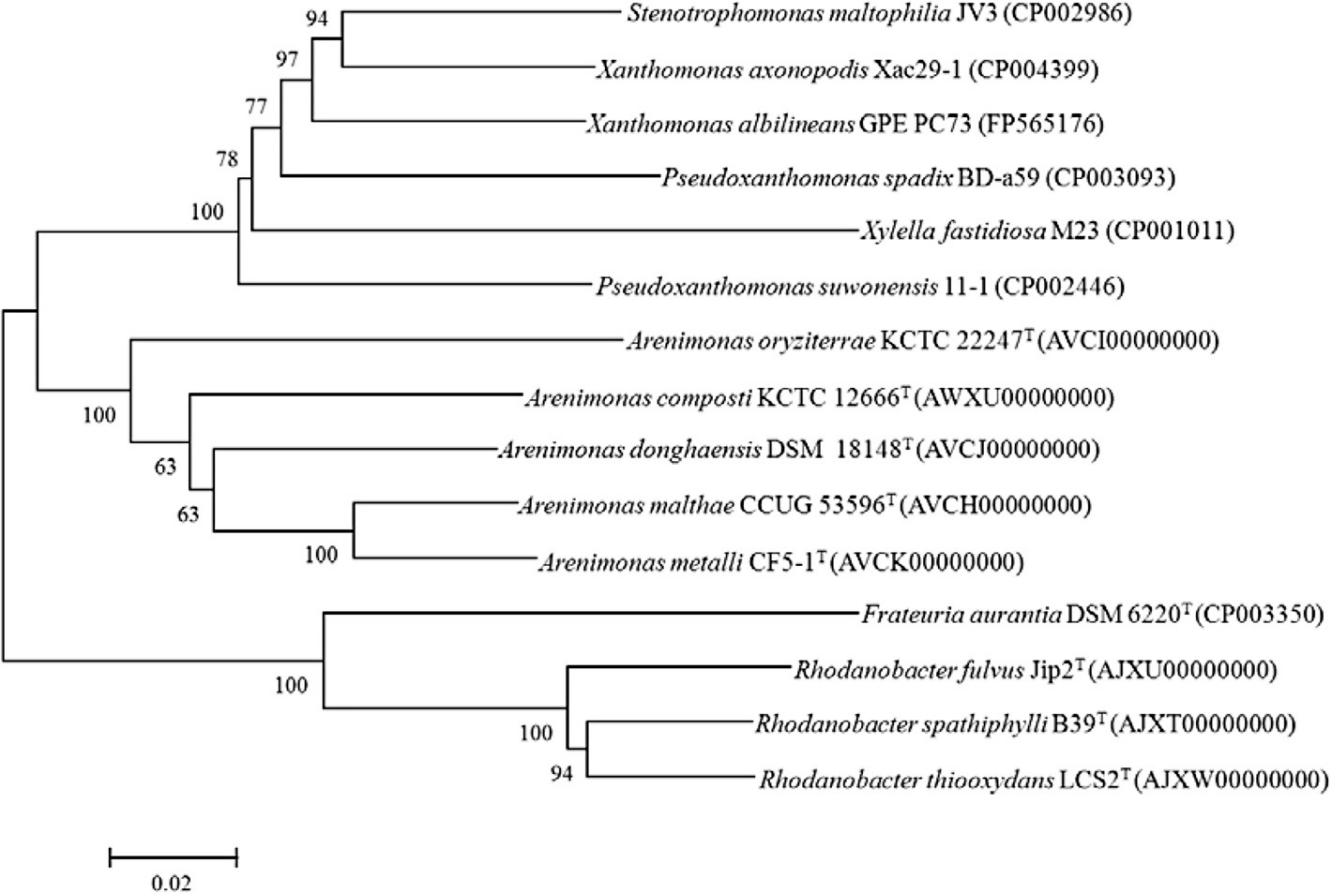
A phylogenetic tree highlighting the phylogenetic position of *A. donghaensis* DSM 18148^T^. The conserved protein was analyzed by OrthoMCL with Match Cutoff 50 % and E-value Exponent Cutoff 1-e^5^ [15]. The phylogenetic tree was constructed based on the 1014 single-copy conserved proteins shared among the fifteen genomes. The phylogenies were inferred by MEGA 5.05 with NJ algorithm [8], and 1000 bootstrap repetitions were computed to estimate the reliability of the tree. The genome accession numbers of the strains are shown in parentheses

Fifteen available genome sequences of the family *Xanthomonadaceae* were chosen for genomic based phylogenetic analysis, including the five *Arenimonas* genomes that were sequenced by us. In total, 1014 core protein sequences were extracted using the cluster algorithm tool OrthoMCL with default parameters [15]. The neighbor-joining (NJ) phylogenetic tree showed that the five *Arenimonas* species clustered into the same branch (Fig. 5), which is in accordance with the 16S rRNA gene-based phylogeny (Fig. 1).

Similar to *A. donghaensis*DSM 18148^T^, the TCA cycle is complete and hexokinase is absent in all the five *Arenimonas* strains. The proteins responsible for the oxidative phase of pentose phosphate pathway are also incomplete in five *Arenimonas* strains, this may be part of the reasons that the five *Arenimonas* strains can only use several single carbon sources.

## Conclusions

To the best of our knowledge, this report provides the first genomic information of the genus *Arenimonas*. The genomic based phylogeny is in agreement with the 16S rRNA gene based one indicating the usefulness of genomic information for bacterial taxonomic classification. Analysis of the genome shows certain correlation between the genotypes and the phenotypes especially on utilization of sole carbon sources.

## Abbreviations

KACC: Korean Agricultural Culture Collection
DSMZ: German Collection of Microorganisms and Cell Cultures
DPG: Diphosphatidylglycerol
PG: Phosphatidylglycerol
PE: Phosphatidylethanolamine
